# Current Concepts on Genetic Aspects of Mitochondrial Dysfunction in Amyotrophic Lateral Sclerosis

**DOI:** 10.3390/ijms22189832

**Published:** 2021-09-11

**Authors:** Milena Jankovic, Ivana Novakovic, Phepy Gamil Anwar Dawod, Ayman Gamil Anwar Dawod, Aleksandra Drinic, Fayda I. Abdel Motaleb, Sinisa Ducic, Dejan Nikolic

**Affiliations:** 1 Neurology Clinic, Clinical Center of Serbia, 11000 Belgrade, Serbia; milena.jankovic.82@gmail.com; 2 Faculty of Medicine, University of Belgrade, 11000 Belgrade, Serbia; ivana.novakovic@med.bg.ac.rs (I.N.); fibygamilanwer@med.asu.edu.eg (P.G.A.D.); a.drinic@hotmail.com (A.D.); sinisa.ducic@udk.bg.ac.rs (S.D.); 3 Department of Medical Biochemistry and Molecular Biology, Faculty of Medicine, Ain Shams University, 11591 Cairo, Egypt; dr.fayda@hotmail.com; 4 Internal Medicine, Hepatogastroenterology and Endoscopy Department, Faculty of Medicine, Ain Shams University, 11591 Cairo, Egypt; ayman.gamil@med.asu.edu.eg; 5 Pediatric Surgery Department, University Children’s Hospital, 11000 Belgrade, Serbia; 6 Physical Medicine and Rehabilitation Department, University Children’s Hospital, Tirsova 10, 11000 Belgrade, Serbia

**Keywords:** amyotrophic lateral sclerosis, genes, mitochondria, biomarkers, therapy

## Abstract

Amyotrophic Lateral Sclerosis (ALS), neurodegenerative motor neuron disorder is characterized as multisystem disease with important contribution of genetic factors. The etiopahogenesis of ALS is not fully elucidate, but the dominant theory at present relates to RNA processing, as well as protein aggregation and miss-folding, oxidative stress, glutamate excitotoxicity, inflammation and epigenetic dysregulation. Additionally, as mitochondria plays a leading role in cellular homeostasis maintenance, a rising amount of evidence indicates mitochondrial dysfunction as a substantial contributor to disease onset and progression. The aim of this review is to summarize most relevant findings that link genetic factors in ALS pathogenesis with different mechanisms with mitochondrial involvement (respiratory chain, OXPHOS control, calcium buffering, axonal transport, inflammation, mitophagy, etc.). We highlight the importance of a widening perspective for better understanding overlapping pathophysiological pathways in ALS and neurodegeneration in general. Finally, current and potentially novel therapies, especially gene specific therapies, targeting mitochondrial dysfunction are discussed briefly.

## 1. Introduction

Amyotrophic Lateral Sclerosis (ALS) or Lou Gehrig’s disease belongs to the group of neurodegenerative diseases, where both upper and lower motor neurons are affected [1]. In the review of the Logroscino it was stated that the ALS can be described as multisystem disease with affection of numerous other domains that include: cognitive, behavioral, autonomic system and extrapyramidal motor system [2]. Around 5-10% of diagnosed cases belongs to the familial ALS (fALS) usually with autosomal dominant inheritance. The most common mutations occur in genes *SOD1* (superoxide dismutase-1), *C9ORF72* (Chromosome 9 open reading frame 72), *TARDBP* (TAR DNA/RNA-binding protein of 43 kDa), *FUS* (RNA-binding protein fused in sarcoma), but additional genes with very low mutation frequencies or even private mutations are also described [3,4]. Besides those rare monogenic forms of the disease, in sporadic ALS (sALS), genetic factors express important contribution either through increasing risk or modifying the disease course of ALS, following the common trend in neurodegenerative disorders.

The incidence of ALS ranges between 0.6 and 3.8 per 100,000 person-years, with higher rates in Europe between 2.1 and 3.8 per 100,000 person-years [5], while lower rates were observed in African, Hispanic and Asian populations [6]. With aging, the incidence increases, particularly after 40 years of age [1]. The prevalence rates of ALS range between 4.1 and 8.4 per 100,000 persons [5]. Furthermore, Kadena and Vlamos pointed that it is expected that the population of ALS patients will increase significantly between 2015 and 2040 [6].

Despite decades of ALS drug research, there is still no effective cure, and all available treatments are focused on easing the symptoms, improving the quality of life and prolonging the patient’s life. Focusing on better understanding of disease mechanisms that begin years or even decades before the clinical presentation, as well as identifying potential biomarkers and phenotype modifiers, will lead to (i) earlier diagnosis, (ii) distinguishing several subclinical types of the disease, (iii) better prognostic criteria, (iv) diversification of drug targets and subsequently to (v) wider therapeutic possibilities at early stages and during the disease progression.

In most ALS cases, the disease is defined as TDP-43 proteinopathy, where neurodegeneration is linked to accumulation of cytoplasmic TDP-43 [7]. The exact etiopathogenesis of ALS is not known; the dominant theory at present relates to RNA processing, but protein aggregation and miss-folding, oxidative stress, glutamate excitotoxicity, inflammation and epigenetic dysregulation play important roles also [8]. A number of data indicate mitochondrial dysfunction as a substantial contributor to disease onset and progression.

The quick medical facts about ALS are presented in Table 1.

## 2. Phenotype Variability

The ALS affects both upper and lower motor neurons; however, cases with only upper or only lower motor neuron affection were described [17]. Both motor variability (bulbar dominant, lower motor neuron dominant and upper motor neuron dominant) and extra-motor variability (ALS plus syndrome) were assessed in the study of Takeda et al. [17]. It is noticed that not all motor neurons have the same affinity of affection in ALS. Those with larger diameter are more vulnerable, while oculomotor neurons and those in Onuf’s nucleus are more resistant [18]. The possible explanation for increased vulnerability of larger motor neurons could be found in mitochondrial dysfunction which leads to the increased cytosolic calcium levels that might have the result of the possible motor neuron death, since the larger ones do not contain calcium binding proteins like parvalbumin and calbindin D-28k [19].

Furthermore, it was stated that different mutations in certain genes or different mutated genes can be linked to the ALS phenotype; however, the same mutation in certain genes via pleiotropy can modulate multiple phenotypic characteristics [20]. It is well established that ALS is genetically and pathologically overlapping with frontotemporal dementia (FTD), a neurodegenerative disorder whose presenting symptoms drastically differ from motor neuron diseases [21]. Finally, the effect of oligogenic inheritance was also stressed as a potential contributor to the ALS development [20]. On the other hand, fast progression in next-generation sequencing (NGS) technologies application, not only in the research, but also in the clinical settings, is leading to identification of numerous genetic variants in ALS patients. Generally accepted guidelines for variant interpretation are already established by the American College of Medical Genetics and Genomics (ACMG) [22], but due to specificity and complexity of ALS, it may become challenging to characterize certain variants and to predict possible consequences. Lattante et al. recently addressed that issue and proposed relevance of each ACMG criterion in ALS as well as in which ALS genes criteria are applicable and under what circumstances [23]. Through detailed explanations and suggestions, authors provided the fundamentals for developing standards and guidelines that would be specific for the interpretation of ALS-related variants [23].

Weishaupt at al. pointed out that mutations in different genes are actually affecting several major pathways in the ALS/FTD spectrum. Those processes include RNA metabolism, selective autophagy (including mitophagy), cytoskeleton dynamics DNA reparation etc. and could be functionally united into single “mega-pathway” [24]. It is obvious that, at certain point, mitochondrial network is involved in all of abovementioned mechanisms. In this review, we aimed to highlight that impact and how mitochondrial dysfunction is turning “mega-pathway” into “ALS-pathway”.

## 3. Mitochondrial (Dys)Function

Mitochondria are organelles that have a significant role in eukaryotic cellular metabolism and survival, including adenosine triphosphate (ATP) production, phospholipid biogenesis, calcium homeostasis, calcium signaling and apoptosis [25,26]. About 1500 proteins play a role in different mitochondrial functions; the majority of them are encoded by nuclear genes, but a small proportion is encoded by their own mitochondrial DNA (mtDNA). The morphology and function of these organelles are regulated by fusion, fission and mitophagy, thus any dysregulation within these processes might result in fragmentation, elongation and aggregation of mitochondria [27]. As mitochondrial dynamics are regulated by GTPase dynamin-related proteins, studies suggest that modification of its activity may mediate degeneration of motor neurons in ALS [27,28]. Recent study found that Dynamin-related protein 1 (Drp1) was dephosphorylated by protein phosphatase 1 (PP1), which is pathologically enhanced in primary neuronal culture models, induced pluripotent stem (iPS) cells-derived human motor neurons and zebrafish models of ALS [29].

In ALS neurons, mitochondria were found to be structurally impaired (swollen and vacuolated). Furthermore, mitochondrial dysfunction might have serious negative effects on cells via increased production of reactive oxygen species (ROS), damaging nucleic acids, lipids and proteins [19].

In addition, impaired energy production is one of the consequences of mitochondrial dysfunction, and it severely affects ALS neurons, because neuronal activity and synaptic function are dependent on the energy supply by mitochondrial oxidative phosphorylation (OXPHOS) complexes [30].

Since mitochondria participate in calcium ion homeostasis, increase of calcium ions in cytoplasm leads to the accumulation of such ions in mitochondria as buffering mechanism [26]. This is of great importance since calcium ions participate in signaling pathways which are significant for cell survival [26].

Another important aspect of mitochondrial dysfunction in neural tissue refers to the axonal transport system failure which might be recognized as one of the pathogenic mechanisms in ALS [26]. In neurons there is an active axonal transport of numerous materials, such as proteins and organelles. Both slow and fast axonal transports are described [31]. The fast one that is responsible for mitochondrial transport is mediated by kinesin (anterograde transport) and dynein (retrograde transport) motor complexes [31], thus any disturbances in these motor complexes could alter the neuron function.

## 4. Mitochondrial Genome Variations

More than 20 years ago, Brothwick et al., demonstrated decreased activity of enzyme cytochrome c oxidase in spinal cord motor neurons of ALS patients. Having in mind that enzyme is mtDNA encoded, authors proposed the idea of mutation accumulation in mtDNA induced by oxidative stress, as a possible cause of ALS [32]. As it is mentioned above, mtDNA is coding just a small proportion of more than one and a half thousand of proteins predominantly located in mitochondria. Human mtDNA is about 16kb circular molecule present in multiple copies in each mitochondrion and 103 and 104 copies per cell [33]. Besides 13 protein coding genes, mtDNA contains 22 tRNA and 2 rRNA genes enabling intramitochondrial translation. Stressed mitochondria activate inflammation and innate immunity by releasing mitochondrial damage–associated molecular patterns (DAMPs) and mtDNA into cytoplasm [34,35]. Additionally, biochemical studies noted changes in mtDNA that are either increased mutant forms or reduced amount in spinal cord of sALS patients [19]. Recent studies of Stoccoro et al. showed reduced methylation of the mtDNA regulatory region (D loop), in peripheral lymphocytes in SOD1-fALS and in sALS. This was associated with an increased number of mtDNA copies and interpreted as a compensatory mechanism to counteract mitochondrial impairment in such ALS cases [36,37].

## 5. Genes Involved in Mitochondrial Dysfunction

**Figure 1:**
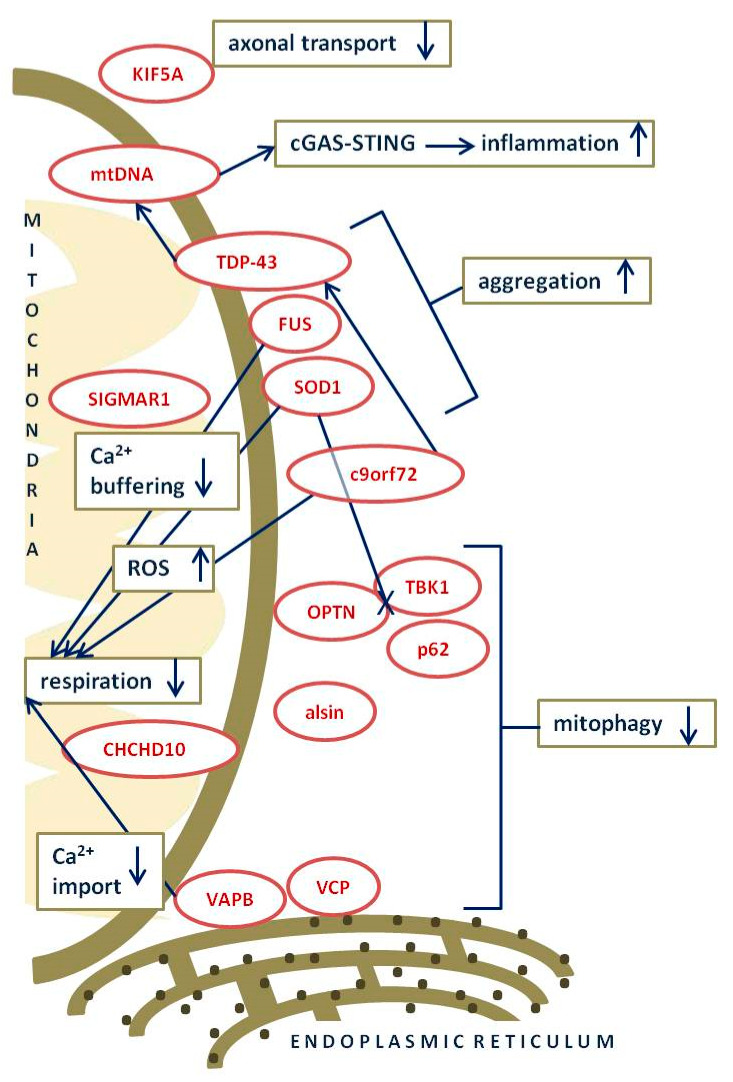
Schematic summary of most prominent ALS–associated mutated genes and their involvement in mitochondrial dysfunction. Dark blue text in squares with green ochre outline–processes affected by ALS–associated mutated genes; dark red text in oval shape with red outline–ALS–associated mutant proteins; dark blue long arrows–connecting ALS–associated mutant proteins with affected processes; up/down arrows–increased/decreased process; dark blue long line with X–impaired interaction of proteins; square black dots–ribosomes. More detailed explanation of particular mechanisms is in the following text.

Mounting evidence is establishing mutations in numerous ALS-genes as cause of mitochondrial dysfunction through impaired respiration, protein import, mitochondrial transport, homeostasis and degradation, and destabilization of mtDNA (Figure 1).

The first gene associated with ALS was *SOD1* and almost 200 different pathogenic mutations have been identified in patients so far [38,39]. SOD1 (superoxide dismutase 1 or Cu-Zn superoxide dismutase) protein is a cytosolic and mitochondrial antioxidant enzyme with primary function in converting superoxide to molecular oxygen and hydrogen peroxide, but due to its unexpected abundance in central nervous system, it is proposed that SOD1 may play other roles [40]. Additionally, different models (mouse or cell lines) with *SOD1* mutations are the most studied models of ALS and it is proposed that mutations cause gain of toxicity although the mechanism is not completely understood [39]. Mutant SOD1 is making aggregates locate at the surface of outer mitochondrial membrane and reducing outer mitochondrial membrane permeability [41]. Those aggregates are directly inhibiting respiration, increasing oxidative stress and cause mitochondrial damage and cytohrome c release [41]. Novel study demonstrated that mutant SOD1 aggregates affect mitophagy by disabling recruitment of autophagy receptors on damaged mitochondria [42].

*C9ORF72* gene mutation has been recognized as the most commonly known genetic cause of ALS. In 2011 two independent groups published breakthrough results about *C9ORF72* non-coding hexa-nucleotide repeat expansion (GGGGCC or G4C2) in ALS and Frontotemporal Dementia (FTD) cases, providing the first molecular link between these two disorders [43,44]. In that moment gene *C9ORF72* (Chromosome 9 open reading frame 72) and corresponding protein C9ORF72 were completely unknown; now, 10 years later, our knowledge is substantially expanded. The G4C2 repeat sequence is located in the first intron of the *C9ORF72* gene, with less than 30 repeats in healthy subjects. Small proportion of patients shows expansion of 70 to 100 units, and the majority of them have hundreds and thousands of repeats. *C9ORF72* expansion is detected in both familial and sporadic cases, with prevalence of up to 40% of fALS, and up to 7% of sALS, depending on the studied population [45], and frequent ALS/FTD overlaps. Patients are heterozygous for repeat expansion and mutation exhibits dominant characteristics with age related penetrance [45]. As expected in repeat disorders, somatic mosaicism in repeat number is observed, but anticipation phenomenon is not clearly confirmed. Regarding pathogenic mechanisms, the non-coding G4C2 expansion has both loss-of-function and gain of function effects. Loss of function causes functional C9ORF72 haploinsufficiency. On the contrary, gain of function is the result of expression of abnormal bidirectionally transcribed RNAs with the repeat, with neurotoxicity of RNA focuses and poly(GR) peptides produced in repeats RAN (repeat associated non AUG) translation. It has been shown that C9ORF72 poly(GR) aggregation induces TDP-43 inclusions, in an RNA independent manner [46]. According to the synergistic model, both C9ORF72 loss of function and gain of function contribute to pathogenic disease phenotype [45]. However, the exact molecular mechanism determining ALS or FTD or overlapping ALS/FTD phenotype is not known. *C9ORF72* is expressed mainly in neurons and in glial cells and in myeloid cells as well. Studies show C9ORF72 is an important modulator of the activity of small GTPases that regulate membrane trafficking, autophagy and axonal and synaptic maintenance. C9ORF72’s role in mitochondria has been elucidated recently. A link between C9ORF72 and mitochondria dysfunction was assessed for the first time in the study of Onesto et al. [47]. In fibroblasts of C9-ALS patients they showed gene-specific mitochondrias’ changes in oxidative conditions. They speculated that, together with *TARDBP, C9ORF72* mutations might trigger death of ALS neurons by affecting not only RNA metabolism but also mitochondria activity. Lopez-Gonzalez et al. studied human motor neurons derived from C9ORF72 ALS iPS cells. They showed production of reactive oxygen species and increased mitochondrial potential as well as high levels of DNA damage with activation of the p53 pathway [48]. Analysis of poly(GR) interactome discovered a number of mitochondrial ribosomal proteins in network, which confirmed poly(GR) preferential binding to the mitochondria and oxidative stress induction. Li et al. suggested that poly(GR) can be translated in close proximity to the mitochondrial surface; stalling of its translation triggers ribosome-associated quality control and C terminal extension with potentially toxic aggregations of poly(GR) on mitochondria [49].

Dafinca et al. reported reduced mitochondrial Ca^2+^ buffering capacity and reduced membrane potential in motor neurons derived from iPS cells of C9ORF72-ALS patients [50,51]. Interestingly enough, such effects are reversed by genome editing using CRISPR/Cas9-mediated homologous recombination indicting *C9ORF72* could be a target for gene therapy. A very recent study by Mehta et al. showed abnormalities in the electron chain machinery in the same cell model, with detected low levels of basal respiration and maximal mitochondrial respiration [52]. Observed energetic reduction was correlated with lowered expression of complexes I and IV of the mitochondrial electron transport chain. Results of a very recent study by Wang et al. are in agreement with these findings [53]. They demonstrated that C9ORF72 regulates energy homeostasis by stabilizing mitochondrial complex I (CI) assembly. C9ORF72 is imported into mitochondrial intermembrane space by AIFM1 (apoptosis inducing factor -mitochondria associated 1), and then mitochondrial C9ORF72 is crucial for OXPHOS functions and energy metabolism. C9ORF72 enables effective CI assembly by stabilizing complex I assembly factor TIMMDC1 (translocase of inner mitochondrial membrane domain containing 1) and also interacts with PHB (prohibin) complex, another important chaperone for CI and other mitochondrial functions. In the absence of C9ORF72 the level of mature CI and CI activity was reduced. This result has been confirmed in iPSC-derived motor neurons from C9ORF72-ALS patients [53].

In addition, Choi et al. studied toxic effects of C9ORF72 poly(GR) in established (GR)80 mouse model. They showed that pathological products bind preferentially to the mitochondrial complex V component ATP5A1 and compromise mitochondrial function in vivo in a time dependent manner [54]. This is consistent with observed decreased ATP5A1 protein level in both (GR)80 mouse neurons and patient brains. Such poly(GR)-induced neurotoxicity cold be reduced by ectopic Atp5a1 expression or decreasing poly(GR) level in mouse model. Authors concluded that poly(GR)-induced mitochondrial defects are a major driver of disease initiation in C9ORF72-related ALS/FTD.

These results reveal a previously unknown function of C9orf72 in mitochondria and suggest that defective energy metabolism may underlie the pathogenesis of relevant diseases.

Other major contributors to the number of ALS cases are mutations in genes *TARDBP* and *FUS*, both coding DNA/RNA binding proteins involved in, amongst other mechanisms, mitochondrial impairment.

TDP-43 protein, coded by gene *TARDBP*, has predominantly nuclear localization but also contains specific sequence for transport to cytoplasm. Cytoplasmic TDP-43 is increasing proinflammatory cytokine production through activation of two pathways (nuclear factor κB (NF-κB) and type I interferon) [55,56]. As it was mentioned, neuropathological hallmark of the disease in the vast majority of ALS patients are TDP-43 aggregates in the cytoplasm of motor neurons [7]. On the other hand, numerous missense mutations in gene *TARDBP* are provoking TDP-43 accumulation in mitochondria [57]. Possible crucial connection of TDP-43, mtDNA and inflammation in pathogenesis of ALS has been demonstrated recently in a remarkable study by Yu at al. Using motor neurons derived from induced pluripotent stem cell (iPSC) of patients with ALS, authors proved previous findings that TDP-43 enters mitochondria via TIM22, the mitochondrial import inner membrane translocase, and destabilizes homeostasis. Subsequently, it triggers the opening of the mitochondrial permeability transition pore (mPTP) and the release of mtDNA into the cytoplasm. Cytosolic mtDNA is recognized as an invading agent by cyclic GMPAMP synthase (cGAS) and binding mtDNA with cGAS is activating the stimulator of interferon genes (STING). Further support and clarification of these findings is provided in the mouse model of ALS with human TDP-43 (A315T) overexpression and post-mortem spinal cord samples of patients with sporadic ALS [58]. cGAS–STING pathway regulates the type I interferon and other inflammatory cytokines, including tumor necrosis factor-α (TNF-α), and provides fast-acting innate immune response to cytosolic DNA [59,60]. The similar role of STING is observed in other neurodegenerative and autoimmune diseases. Sliter et al. have recently showed in a PD mouse model that PINK1 and Parkin, mitochondrial quality control regulators, facilitate STING-induced inflammation and that loss of dopaminergic neurons and neurodegeneration can be rescued by loss of STING [61].

Another member of DNA/RNA binding proteins (RBPs) associated with ALS is FUS. Mutations in gene *FUS* cause ALS with earlier onset of first symptoms and faster disease progression. The exact pathological mechanism is still elusive, but it is proposed that the mutated protein probably develops a novel, toxic function. Additionally, the most common mutations primarily affect nuclear localization signal domain and, subsequently, a fraction of mutant protein becomes located in the cytosol [62]. Similar to other RBPs, aggregates containing mutant FUS have been detected in motor neurons of ALS patients. It has been shown that mutant FUS in mouse model targets mRNAs that are encoding proteins of mitochondrial respiratory chain and mRNAs encoded by the mitochondrial genome. Subsequently, the mitochondrial network becomes disrupted, respiration is reduced, and ROS production is increased. These findings are further confirmed in transcriptomic analyses, but association of FUS with mitochondrial mRNA was not proved as ALS pathology mechanism [63,64]. Among proteins recognized as targets for mutant C9orf72, TDP-43 or FUS are valosin containing protein (VCP) and vesicle-associated membrane protein-associated protein B (VAPB). Interestingly, those proteins, coded by ALS-associated genes *VCP* and *VAPB*, are responsible for normal functioning of mitochondria-endoplasmic reticulum contacts (MERCs), structures that are controlling mitochondrial metabolism and oxidative stress level [65].

VAPB protein is involved in lipid and calcium ion transfers between endoplasmic reticulum and mitochondria and mutant VAPB is compromising mitochondrial functioning through reducing OXPHOS due to insufficient Ca^2+^ import in mitochondria [66]. *VAPB* mutations are described in autosomal dominant ALS as well as in late-onset spinal muscular atrophy [67] and recent study pointed out that dysfunctional autophagy may be one of the pathomechanisms provoked by common mutation in gene *VAPB* [68].

Supporting evidence for inefficient mitochondrial clearance as underlying disease mechanism are mutations in gene *VCP* (valosin-containing protein), described in ALS and FTD patients [69,70]. VCP protein, ubiquitin-specific chaperone, is identified as an essential player in mitochondrial quality control and mutant VCP is disturbing mitochondria labeling for mitophagy [71].

As mitophagy is a process of selective autophagy and damaged mitochondria are subjected to degradation in order to maintain high functionality of mitochondrial network, it is not surprising that a rising number of ALS-associated genes are coding proteins involved in the mitophagy. The process of mitophagy is PINK1/Parkin mediated and requires recruitment of autophagy receptors like optineurin (OPTN) and mutations in gene *OPTN* are first described in patients with glaucoma and later in ALS patients [72]. After Parkin initiated ubiquitination of mitochondria, recruited OPTN induces autophagosome formation [73]. It has been demonstrated that certain *OPTN* mutations significantly decrease recruitment of OPTN to damaged mitochondria, as well as mutant SOD1 [42,73].

Gene *TBK1* codes TANK-binding kinase 1 (TBK1), a protein involved in innate immunity, but that is also responsible for phosphorylation of OPTN during preparation of depolarized mitochondria for autophagosome formation. Interestingly, mutations in gene *TBK1* were also first associated with two types of glaucoma and then with ALS [74,75]. Mutations in both *OPTN* and *TBK1* genes are disturbing selective autophagy and subsequently reduce adequate clearance of mitochondria [76]. Association of mutations with ALS suggests that compromised mitophagy and subsequently damaged mitochondria accumulation may be underlying disease mechanisms. These findings are further confirmed by functional study in transfected HeLa cells with mutated *OPTN* and *TBK1*. Using live-cell assays, authors have shown that mutations are preventing autophagosome creation [73].

Functionally related to TBK1 is also p62 protein coded by gene *SQSTM1*. Different types of mutations in *SQSTM1* are associated with several different phenotypes; missense mutations are described in ALS, while truncating mutations are more prevalent in autosomal dominant bone diseases and autosomal recessive, young onset, progressive neurodegeneration [77,78,79]. Sequestosome 1/p62’s main function is to facilitate protein degradation, conducted through ubiquitin-proteasome system or autophagy. Mutant protein is disturbing those systems and subsequently leading to an increased number of dysfunctional mitochondria [80].

Additionally, mitophagy and numerous mitochondrial and Golgi apparatus functions are found disturbed in the absence of protein alsin, although the precise molecular role of this protein is not fully elucidated [81]. Mutations in gene *ALS2*, coding for alsin protein, are also associated with rare juvenile ALS with autosomal recessive inheritance and these findings contribute to impaired mitochondrial clearance as a disease mechanism [82,83].

Sigma non-opioid intracellular receptor 1 (SIGMAR1) is another MERC-associated chaperon, responsible for maintaining calcium signaling [65]. Mutations in gene *SIGMAR1* are described in autosomal recessive juvenile ALS, as well as in distal neuropathies [84]. Loss of SIGMAR1 function is causing mitochondria-associated membrane breakdown and reduction of metabolic signaling and ATP production, as a pathomechanism of ALS [85].

*CHCHD10* (coiled-coil-helix-coiled-coil-helix domain containing 10) mutations are first described in late onset ALS and FTD with slow disease progression [86]. CHCHD10 is a small soluble protein with predominantly mitochondrial intermembrane space localization. The exact molecular function is still unknown as well as how mutations in this gene are associated with ALS, but homeostasis of mitochondrial membrane proteins is proposed [87]. Recent study identified a novel mutation (Q108P) with atypical, young onset and aggressive course ALS. Further studies suggested that mitochondrial import of mutant CHCHD10 is almost completely inhibited and that over expression of Mia40, a protein involved in redox system for protein import, may rescue the phenotype [88]. These findings address several very important issues (i) importance of CHCHD10 for mitochondrial respiration in motoneurons (ii) confirmation of *CHCHD10* mutations association with ALS and FTD spectrum (iii) recognition of mitochondrial protein import impairment as possible pathogenic mechanism in disease progression (iv) development of “epigenetic boosting” therapies targeting expression of proteins involved mitochondrial protein import machinery and/or CHCHD10 (to overcome haploinsufficiency).

It is important to note that overexpression of TDP-43 leads to nuclear localization of CHCHD10 and it is observed that the protein affects transcription [89]. Development of therapies that increase expression may provoke wider effects on transcription machinery and should be carefully balanced.

One of the genes most recently found to be associated with ALS is *KIF5A*, coding for motor protein, kinesin family member 5A [90,91]. It is interesting that this gene is expressed selectively in neurons and mutations are previously described in other neurological diseases, even in neonatal developmental syndromes. KIF5A is involved in organelle transport, including transport of mitochondria and mutations are already associated with mitochondrial dysfunction [92]. Disrupted mitochondrial transport to different cell parts with higher metabolic activity and/or with need for regulation of calcium ions homeostasis leads to cell damaging and inflammation. Linkage of all these findings draws attention to transport of mitochondria as one of the pathological mechanisms that are possibly contributing to ALS pathogenesis.

## 6. Novel Biomarkers and Therapies for ALS: All Roads Lead to Mitochondria?

Majority of proposed mechanisms occur very early in pathological process of disease development and before clinical presentation of ALS, putting mitochondrial dysfunctions in central focus of predictive, prognostic and therapeutic research.

Involvement of microRNAs (miRs) and long non-coding RNAs (lncRNAs) in different neurodegenerative disorders was under intense research in recent decades. MicroRNAs (miRs), regulatory molecules that affect translation of mRNA targets, are established as potent biomarkers in numerous diseases and it is not unexpected that miRs are also differently expressed in ALS patients. Several of differently expressed miRs are controlling expression of genes involved in oxidative stress regulation and may be considered as biomarkers and/or therapeutic targets for ALS (miR-27a, miR-34a, miR-155, miR-142-5p, and miR-338-3p) [93]. MiR-338-3p is regulating nuclear mRNA coding for mitochondrial subunits of the oxidative phosphorylation machinery [94]. On the other hand, oxidative stress is affecting expression of numerous miRs and mitochondrial impairment is one of the main sources of oxidative stress, creating a vicious circle of the disease. Recent study established miR-124 as a new therapeutic target as well as potential biomarker in ALS patients [95]. Mir-124 upregulation is disturbing mitochondrial axonal transport through negative regulation of vimentin, filament that controls position and metabolic activity of mitochondria [96]. Disruption of mitochondrial network in skeletal muscles is affecting ALS progression and miR-23a, miR-29b, miR-206 and miR-455 increased skeletal muscle of ALS patients compared to controls [97]. MiR-23 reduces PGC-1alpha signaling, which is involved in mitochondrial biogenesis. These findings propose that drug inhibition of miR-23a may rescue PGC-1alpha function and slow down disease progression [97]. The importance of lncRNAs in ALS pathogenesis has b een recently recognized and several antisense (AS) lncRNAs were found differentially expressed in ALS patients. In FUS-ALS the PAX antisense was differentially expressed in TARDP-ALS SNAP25-AS and in SOD1-ALS CKMT-22-AS. In sporadic ALS the number was much higher and UBXN7-AS was associated with mitochondrial function. UBXN7-AS is regulating a ubiquitin protein, UBXN7, bonded with previously mentioned VCP [98].

Similar to miRs and lncRNAs, RNA binding proteins regulating mRNA life cycle and their dysfunction, implicated in oxidative stress along with the mitochondrial dysfunction in ALS, has b een reviewed recently [99]. It is interesting to point out that ALS-related mutations are detected in several genes coding for RBPs including previously mentioned *TARDBP* and *FUS*. The finding that mutant FUS shows toxic gain of function by interacting with mitochondrial respiratory chain components and mRNAs encoded by the mitochondrial genome established FUS as a potential target for autophagy, stimulating drug development [63,64]. Novel study, addressing effects of *FUS* mutations on RBPs misregulation, showed that drug induced autophagy restores homeostasis [100]. These results could be translated to other ALS subtypes and development of drugs that would induce mitophagy if it is impaired.

The current treatments for ALS are only possible to improve a little quality of life and/or to decrease the disease progression rate and prolong disease duration. Lack of neuroprotective and efficient therapy for ALS is major concern and leading force in numerous studies and mitochondrial impairment is identified as one of the potential targets for novel drugs. Effects in modulating the permeability of mitochondrial transition pore were investigating olesoxime [101,102,103] and GNX4728 [104]. Although some animal studies showed the improvement and increased survival in ALS mice, the drugs were not translated to patients. Another potential drug that demonstrated reduction of neuronal cell death in ALS mice is a neuronal sigma-1-receptor agonist (SA4503). This agonist reduces oxidative stress and regulates calcium flux [105]. Clinical trials for combination of two drugs, dextromethorphan and quinidine, that are affecting demethylation of P450 cytochrome enzyme, are showing improvement of speech and swallowing in ALS patients [106,107].

Focusing on mitochondrial function and ROS, several candidates have been tested. A free radical scavenger Edaravone has been approved by the FDA for the treatment of early-stage ALS patients after showing up to slow down the functional decline. However, its definitive efficacy is still under debate. It has been observed in animal models that Edaravone protects neuronal cells, at least in part, by maintaining mitochondria [108,109]. Coenzyme Q10 or Ubiquinone seems another good candidate for ALS drug. As it is well known, Coenzyme Q10 plays a central role in mitochondrial oxidative phosphorylation and the production of ATP; it also functions as an antioxidant in cell membranes and lipoproteins. However, this compound was not proven effective, although some clinical trials are still ongoing. Several other molecules acting on mitochondrial dysfunction were tested without definitive positive results [110,111].

Gene-specific therapies are identified as promising treatments for patients with mutations in ALS associated genes. As toxic gain of function is the most likely mechanism in ALS patients with *SOD1* mutations, potential therapeutic effect would be reducing concentration of mutant protein. Most recent studies and clinical trials are focusing on development of drugs based on non-viral approach with antisense oligonucleotides (ASOs) for gene specific therapies in ALS. Animal studies with ASO for Sod1 mRNA already reported potentially promising results (prolonged survival and better motor functions) [112]. These results encouraged clinical trial (phase 1 and 2) of ASO drug tofersen. The study was designed to investigate the effect of different drug doses and placebo on SOD1 level in cerebrospinal fluid. Despite several limitations, as small number of patients and different, mutation specific clinical presentation and disease progression rate, reduction of SOD1 protein concentration was significant and dose dependent [113]. In addition, ASOs and small interfering RNA (siRNA) were confirmed as efficient in pre-clinical models of ALS with *C9ORF72* and *FUS* mutations, and various clinical trials using this approach are ongoing [110,111,114]. One of the important disadvantages of this strategy is short lifespan of RNA molecules and then necessity of repeated administration. The use of viral vectors expressing ASO or siRNA, in particular adeno-associated viral (AAV) vectors with neural tropism, should overcome that issue and enable long term expression after one single administration.

Studies of potential drugs targeting mitochondrial impairment are presented in Table 2.

Recent discoveries of novel pathological pathways in ALS pointed out alternative directions in developing therapies for not only slowing down but also stopping progression of the disease, especially in early stages. In previously mentioned study, Choi et al. proved that prevention of Drp1 activity is reducing neurodegeneration and that PP1 is involved in dephosphorylation of Drp1 in ALS mice (SOD1 G93A). Furthermore, authors confirmed that inhibition of PP1 restores mitochondrial respiratory functions and rescues neurons from ALS pathology in vivo and in a human iPSC model. Based on those findings, modulation of PP1-Drp1 activity is proposed as a therapeutic target for different features of ALS [29]. Another rising and promising therapeutic possibility is shutting down cGAS-STING cascade. As Yu at al. have proved, genetic deletion or pharmacological inhibition of STING mitigates disease and extends survival in an ALS mouse model and reduces inflammation in iPSC-derived motor neurons from patients with ALS [58]. It is of special importance because ample research and clinical studies are already focused on STING upregulation and development of STING agonists in cancer treatments [115,116]. Those pharmaceutical interests also facilitate programs for therapeutic STING inhibition which may be boosted by recent discovery of small-molecule STING antagonists and efficient blocking of cytokine production in human and mouse cells [117]. Future research will be definitively focused on further understanding the role of cGAS-STING signaling in ALS, as well as in Parkinson’s disease and other neurodegenerative and autoimmune disorders in order to translate STING antagonists from laboratory research to the patients. One of the main goals of novel therapies would be to target the crossroad with the most heavy traffic in neurodegeneration in order to provide wider application for diseases with overlapping phenotypes and pathways.

In addition, most recently created CRISPR-Cas9 mediated genome editing could be applied on SOD1-ALS models, as well as previously mentioned C9ORF72. Because genome editing enables correction of targeted mutation, this methodology is denoted as “the holy grail” of gene therapy, but a number of practical issues should be solved before its clinical application [110,111].

## 7. Conclusions

**Figure 2:**
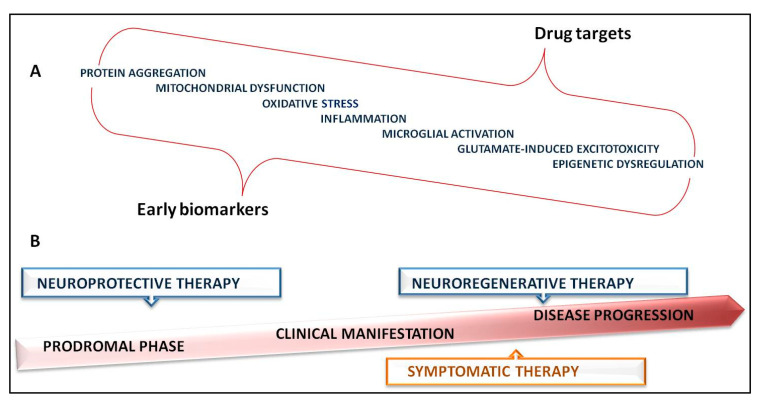
Complex nature of ALS pathogenesis and therapy strategies. (**A**)—“ALS mega-pathway”: pathogenic mechanisms crucial for biomarkers and drug targets identification; (**B**)—therapeutic possibilities timeline.

In summary, with the increasing number of discoveries about mitochondrial involvement in ALS, we can effortlessly conclude how complex the nature of ALS pathogenesis is (Figure 2). Moreover, mitochondrial dysfunction is associated with wide spectrum of clinical presentation and partial overlapping with other neurodegenerative diseases. Multiple pathomechanisms cross through different layers of mitochondrial impairment suggesting that targeting several pathways would be a more promising approach rather than monotherapy. Additionally, the disease phase and activity may be relevant for choosing the right target and the drug because it is well established that during the neurodegeneration predominant pathological pathways are impermanent. Having that in mind, in parallel with developing the therapies, discovering and validating clinically relevant biomarkers of the disease progression and particularly of early, preclinical stages would be of great importance. Mutations in several genes have been proved as ALS causing, providing invaluable possibilities for targeted therapies. On the other hand, rapid biotechnological advances are making a broad range of genetic testing easily accessible for patients, further empowering transfer of novel genetic based therapies to clinical practice.

## Figures and Tables

**Table 1:** Amyotrophic lateral sclerosis quick facts.

Amyotrophic lateral sclerosis		References
**Epidemiological facts**	Prevalence between 4.1 and 8.4/100,000 persons More frequent in males, while male:female ratio nearly equals above 65 years of age Peak between 60 and 75 years of age	[5,9,10,11,12]
**Clinical presentation**	Differ among patients due to the degree of UMN and LMN involvement Bulbar onset (about 25%) Limb onset (about 70%) Initial respiratory affection (5%) **UMN** affection: spasticity, hyperreflexia, clonus, Babinski sign **LMN** affection: weakness, wasting, fasciculations and decreased muscle tone **Bulbar signs:** spastic dysarthria in UMN dysfunction; and weakness, atrophy and fasciculations of the tongue in LMN dysfunction	[12,13,14,15]
**Type**	Sporadic Familial (5-10% of patients)	[11,15]
**Diagnostics**	Physical examination Electrodiagnostic tests (electroneurography and electromyography) Neuroimaging Laboratory testing Serology tests Genetic testing in positive family history	[12,13,16]

UMN—upper motor neuron; LMN—lower motor neuron.

**Table 2:** Studies of potential drugs targeting mitochondrial impairment.

Potential Drug	Structure	Target	Proposed Mechanism	Outcome	Ref.
**Olesoxime**	SM	Mitochondrial permeability	Inhibiting permeability of mPTP	Failure in 18-month survival clinical study (phase 2/3)	[101,102,103]
**GNX-4728**	SM	Mitochondrial permeability	Modulating permeability mPTP	Efficient disease modifying in a preclinical ALS mouse model	[104]
**Cutamesine (SA4503)**	SM	sigma-1 receptor	Control of Ca^2+^ release from ER into mitochondria	Reducing oxidative stress and regulating calcium flux	[105]
**Nuedexta (dextromethorphan and quinidine)**	SM	sigma-1 receptor and P450 cytochrome	Control of Ca^2+^ release from ER into mitochondria	Improving bulbar physiology and function in ALS (approved and recruiting novel trials)	[106,107]
**Edaravone**	SM	ROS	free radical scavenger	Approved	[108,109]
**Tofersen (BIIB067)**	ASO	Mutant SOD1	Reduces levels of mutant SOD1	Ongoing clinical trials phase 1	[113]
**BIIBO78**	ASO	Mutant C9orf72	Reduces levels of mutant C9orf72	Clinical trial phase 1	[114]

SM—Small molecule, ASO—antisense oligonucleotide, mPTP—mitochondrial permeability transition pore, ROS—reactive oxygen species
